# Antibiotic Resistant and Biofilm-Associated *Escherichia coli* Isolates from Diarrheic and Healthy Dogs

**DOI:** 10.3390/microorganisms9061334

**Published:** 2021-06-19

**Authors:** Lívia Karahutová, René Mandelík, Dobroslava Bujňáková

**Affiliations:** 1 Institute of Animal Physiology, Centre of Biosciences of the Slovak Academy of Sciences, Šoltésovej 4-6, 040 01 Košice, Slovakia; karahutova@saske.sk; 2 Department of Epizootiology, Parasitology and Protection of One Health, University of Veterinary Medicine and Pharmacy in Košice, Komenského 73, 040 01 Košice, Slovakia; rene.mandelik@uvlf.sk

**Keywords:** *E. coli*, dogs, antimicrobial resistance, biofilm, phylogenetic groups

## Abstract

Bacteria isolated from companion animals are attracting concerns in a view of public health including antimicrobial resistance and biofilm development, both contributing to difficult-to-treat infections. The purpose of this study was to evaluate the minimum inhibitory concentrations (MIC) of 18 antibiotics in *Escherichia coli* isolated from two groups of dogs (healthy and diarrheic). Isolates were classified into phylogroups, examined for the presence of resistance genes and biofilm-formation capacity. In healthy dogs, phylogenetic analysis showed that 47.37% and 34.22% of *E. coli* isolates belonged to commensal groups (A; B1) in contrast to diarrheic dogs; 42.2% of isolates were identified as the B2 phylogroup, and these *E. coli* bacteria formed a stronger biofilm. The results of healthy dogs showed higher MIC levels for tetracycline (32 mg/L), ampicillin (64 mg/L), ciprofloxacin (8 mg/L) and trimethoprim-sulphonamide (8 mg/L) compared to clinical breakpoints. The most detected gene encoding plasmid-mediated resistance to quinolones in the healthy group was *qnr*B, and in dogs with diarrhea, *qnr*S. The resistance genes were more frequently detected in healthy dogs. The presence of the integron *int1* and the transposon *tn3* increases the possibility of transfer of many different cassette-associated antibiotic-resistance genes. These results suggest that dogs could be a potential reservoir of resistance genes.

## 1. Introduction

*Escherichia coli* (*E. coli)* is a highly versatile bacterium that ranges from harmless gut commensal to intra- or extra- intestinal pathogens [1]. Commensal *E. coli* colonizes in the gastrointestinal tract within a few hours after birth. Although these strains are part of the normal microbiota of humans and animals, several clinical reports have implicated *E. coli* as the etiological agent of diarrhea in humans and their companion animals [2,3]. Previously, the most extensive investigations of *E. coli* infection have been described in cattle, sheep and pigs. However, recently, the dogs and cats that live in close proximity to humans have become a focus of disease transmission studies. Because the contact between humans and pets has increased, the possibility of pathogenic microorganism transmission between these organisms is very high. The fecal shedding of *E. coli* by companion animals represents an important source of the zoonotic transmission of pathogenic agents [2].

The prevalence of drug-resistant bacteria, caused among other things by an excessive use of antibiotics, is an increasing problem due to the possible transmission of resistant bacteria or their resistance genes between animals and humans via direct or indirect contact, such as through food/feed and the environment. Drug-resistant commensal *E. coli* isolates may constitute a significant reservoir of antibiotic-resistance determinants, which can spread to those bacteria that are pathogenic for animals and/or humans. Another problem is biofilm development, since the biofilm matrix gives an additional resistance power to the bacteria which makes them not only tolerant to harsh conditions but also resistant to antibiotics. This leads to the emergence of bad-bugs infections, such as multi-drug resistant, extensively drug resistant and totally drug resistant types of bacteria [4].

For a long time, the focus of research was mostly on antimicrobial resistance (AMR) monitoring in food-producing animals [5]. Recently, dogs and cats have been described as potential vehicles for AMR; however, the data remained scarce. Therefore, it was found necessary to take a closer look at the situation existing in companion animals. Furthermore, approaching any issue from a One Health perspective necessitates looking at the interactions between people, domestic animals including pets, wildlife, plants and our environment [6].

Dogs and cats represent potential sources for the spread of AMR, due to the extensive use of broad-spectrum antimicrobial agents in these animals (even those critically important for human medicine, such as third generation cephalosporins and fluoroquinolones, colistin, tetracyclines and macrolides) and their close and intensive contact with humans [7]. Moreover, pet feces on the ground of urban areas represent a significant public-health problem [8].

So far, especially within the European Union’s member states, the monitoring of the existing situation concerning AMR in indicator bacteria such as *E. coli* of companion animals has been done sporadically. To the best of our knowledge, phenotypic resistance profiles of 282 *E. coli* isolates were determined to be present in dogs and cats in three European countries (Belgium, Italy and the Netherlands), of which 19% were isolated after antibiotic treatment of the monitored animals. Furthermore, the situation in Sweden [9] regards antibiotic resistance in the bacteria from humans and animals (including dogs), and a 331 indicator *E. coli* was mapped in the years 2006 and 2012. A similar situation exists throughout the European Union (EU) including the Slovak Republic, where the data on indicator *E. coli* isolates are only related to poultry and the meat derived thereof. A different situation is presented in the monitoring of AMR in clinical bacteria causing various infections [10]. However, studies of companion animals demonstrating the current situation of AMR concerning the indicator *E. coli* in the EU including the Slovak Republic are rather rare.

Therefore, the objectives of our study were to evaluate the phenotypic and genotypic AMR of commensal indicator and diarrheic *E. coli* isolated from Slovakian canine fecal samples using the standardized automated diagnostic system Bel-MIDITECH to classify their phylogenetic relatedness and to determine their biofilm-forming capacity as one of the factors contributing to increased resilience.

## 2. Materials and Methods

### 2.1. Canine Samples, Isolation and Identification of E. coli

The rectal swabs from 38 healthy non-antimicrobial treated dogs and 45 dogs with diarrhea, of varying breeds and from different households, were inoculated overnight at 37 °C in buffered peptone water (Oxoid, Basingstoke, UK). The samples from the diarrheal dogs were taken before an antibiotic treatment. The samples were then subcultured on MacConkey Agar (Oxoid, Basingstoke, UK) and UriSelect Agar (Bio-Rad Laboratories, Hercules, CA, USA) overnight at 37 °C. The colonies were isolated, identified and confirmed as *E. coli* using the MALDI–TOF MS (Matrix-Assisted Laser Desorption Ionization–Time of Flight, Mass Spectrometry) biotyper (Bruker Daltonics, Bremen, Germany) according to the methods described by Bessède et al. [11] and ENTEROtest24 (Erba Lachema Brno, Czech Republic) for the routine identification of important species of the Enterobacterales family within 24 h. One colony of *E. coli* was isolated from each sample.

### 2.2. Phylogenetic Groups

The form of the phylogenetic analysis was determined by using a new method, according to Clermont et al. [12]. The quadruplex polymerase chain reaction (PCR) was used to determine the phylogroup of each of the 83 isolates corresponding to the presence or absence of the genes *arp*A, *chu*A, *yja*A and *TspE4.C2*. All the isolates assigned to phylogroup A were screened using a C-specific primer *trp*A (trpAgpC). Similarly, all the D phylogroup isolates were screened using an E-specific primer *arp*A (ArpAgpE). The oligonucleotide primers, annealing and references are listed in Table 1.

### 2.3. Antimicrobial Sensitivity

The minimum inhibitory concentration (MIC) testing was performed according to Gattringer et al. [28] using the Slovakian automated diagnostic system Bel-MIDITECH (Bratislava, Slovakia) consisting of ampicillin (AMP), ampicillin + sulbactam (SAM), piperacillin + tazobactam (TZP), cefuroxime (CXM), cefotaxime (CTX), ceftazidime (CAZ), cefoperazone + sulbactam (SPZ), cefepime (FEP), ertapenem (ETP), meropenem (MEM), gentamicin (GEN), tobramycin (TOB), amikacin (AMI), tigecycline (TGC), ciprofloxacin (CIP), tetracycline (TET), colistin (COL) and trimethoprim + sulfonamide (COT). The results of the MIC values of each antibiotic were interpreted according to the clinical breakpoints (CBPs) described by The European Committee on Antimicrobial Susceptibility Testing (EUCAST) 2020 [29].

### 2.4. Detection of Resistance Genes

The strains were investigated for the presence of resistance genes using primers, as shown in Table 1, by means of multiplex and/or single PCR assays. The amplifications were carried out in a single tube with a volume of 25 µL, utilizing TaqI polymerase (Solis Biodyne, Estonia) with 10 × Buffer B without Mg^2+^ (2–2.5 μL); deoxynucleotide triphosphates (dNTPs) mix (Promega, Madison, WI, USA; 2.5 μL); 25 mM MgCl_2_ (1.5–2.5 μL); 10–20 pmol/μL primers (Lambda Life, Bratislava, Slovakia; 0.1–0.2 μL); 10–100 ng/μL DNA template (1–1.5 μL); and deionized sterile water. The PCR program consisted of an initial denaturation step at 95 °C for 4 min, followed by 32 cycles of DNA denaturation at 95 °C for 50 s, primer annealing at 50–69 °C (according to primers) for 50 s and primer extension at 72 °C for 1 min. After the last cycle, a final extension step at 72 °C for 7 min was added. The presence of genes for a resistance to trimethoprim–dihydrofolate reductase enzymes *dfr*A and *dfr*B; sulfonamide resistance–*sul1*, *sul2* and *sul3*; resistance to tetracycline—*tet*A and *tet*B; quinolone resistance–*oqx*A, *oqx*B; additional plasmid mediated quinolone resistance determinants*–aac* (6′)-*Ib-cr*; quinolone extrusion by *qep*A, *qnr*S, *qnr*A and *qnr*B; resistance to colistin encoded by *mcr*-*1* and *mcr*-*2*; β-lactamase encoding *bla*_TEM-1_*, bla*_SHV_ and ampicillinase–cit were monitored. Moreover, we evaluated the presence of genes for integron *int1* and transposon *tn3*, because they are capable of capturing and expressing the genes contained in cassette-like structures that represent a substantial proportion of the resistance determinants in Gram-negative bacteria.

### 2.5. Detection of Biofilm Formation

The ability for biofilm formation was assessed in a quantitative assay using a microtiter-plate test (Nunc, Roskilde, Denmark). Strains were grown on Brain Heart Infusion (BHI) agar, and colonies were re-suspended in a BHI broth (Oxoid, Basingstoke, UK) to reach the 0.5 suspension of McFarland’s standard, and volumes of 200 μL of these cell suspensions were transferred to the wells of the microplate. For the negative control, we used an uninoculated BHI medium. After incubation (24 h at 37 °C), the adherent cells were washed three times using a saline solution and stained with a 0.1% crystal violet solution (Mikrochem, Pezinok, Slovakia). The adhering dye was dissolved with 30% acetic acid, and the optical density was measured at 570 nm in the Synergy HT Multi-Mode Microplate Reader (BioTek, Winooski, VT, USA). For classification, we used the average optical density (OD) value and cut-off value (ODc) (defined as three standard deviations (SD) above the mean OD of the negative control). The final OD value of a tested strain was expressed as the average OD value of the strain reduced by the ODc value. For the interpretation of the results, the strains were divided into the categories described by Stepanovic et al. [30]: OD ≤ ODc = non-biofilm producer; ODc < OD ≤ 2 × ODc = weak biofilm; 2 × ODc < OD ≤ × ODc = moderate and 4 × ODc < OD = strong biofilm producer.

## 3. Results and Discussion

### 3.1. Antimicrobial Sensitivity

A total of 38 *E. coli* isolates recovered from the fecal samples of healthy non-antimicrobial treated dogs and 45 *E. coli* isolates from dogs with diarrhea were investigated to phenotypic and genotypic antimicrobial resistance profiles.

In our study, the highest frequency of resistance in the healthy dogs was recorded for tetracycline (*n* = 13), ampicillin (*n* = 12), ciprofloxacin (*n* = 6), ampicillin + sulbactam (*n* = 6) and trimethoprim + sulphonamide (*n* = 5). This resistance phenotype is the most common, and this could indicate the mobile nature of the genes responsible for these resistance phenotypes [31]. Two of the isolates showed phenotypic colistin resistance.

The group of dogs with diarrhea showed a lower resistance profile for ampicillin (*n* = 0), ampicillin + sulbactam (*n* = 2) and trimethoprim + sulphonamide (*n* = 4). Only their resistance to ciprofloxacin (*n* = 12) and tetracycline (*n* = 14) was higher.

These findings are important for clinicians because β-lactam antibiotics are the most frequently used antimicrobials for gastrointestinal disease in dogs and cats [32]. Ampicillin-resistant *E. coli* could still be isolated from the dogs treated with antibiotics 21 days after treatment [33]. This emphasizes the fact that the intestinal tract acts as a reservoir for resistant bacteria long after the treatment has been stopped. Different studies suggest that high levels of resistance genes can still be found up to four years after antibiotic exposure [34,35]. This once more supports the importance of prudent antimicrobial usage in order to prevent the spread of antibiotic resistance.

### 3.2. Interpretative Reading of the Antibiogram and Detection of Resistance Genes

The most commonly used antimicrobials for companion animals in Europe (e.g., Poland [36], Italy [37], Finland [38], Sweden [39], Norway [40] and the UK [41]) are β-lactams (such as ampicillin, amoxicillin and amoxicillin-clavulanate). Fluoroquinolones, macrolides, tetracyclines, nitroimidazoles and trimethoprim/sulphonamides have been also reported to be routinely used in small animal practice, but on a much smaller scale than β-lactams.

Resistance to ampicillin (AMP) was found in 12 of the *E. coli* isolated from healthy animals. The value of MIC 90 (minimum inhibitory concentration required to inhibit the growth of 90% of microorganisms) for AMP in this group was 64 mg/L. Compared to the EUCAST clinical breakpoint (CBP) (AMP = 8 mg/L), the level of our MIC was very high. Next, a very important antibiotic for this group is ampicillin + sulbactam (SAM), because it has a good safety profile and provides coverage for a wide spectrum of bacterial pathogens. Six isolates from the healthy dogs were resistant to SAM, with a MIC 90 (16 mg/L) value slightly lower than for AMP (CBP for SAM = 8 mg/L), while only two such isolates were found in the dogs with diarrhea (MIC 90 = 8 mg/L). These results are comparable with other studies conducted in Europe [42,43], although a higher resistance is more often reported in southern European countries [10], which supports the importance of detecting the antibiotic profile for success treatment in companion animals. From the β- lactamase genes, we detected only simple *bla*_TEM-1_ in six isolates from the healthy dogs.

Some of the MIC levels found in this study were worrisome. The target MIC value for colistin (COL) is 4 mg/L, or exceptionally 2 mg/L. An interesting finding was the detection of phenotypic colistin-resistance in our two strains from healthy dogs, specifically with values of 4 mg/L and 8 mg/L and one isolate from the dogs with diarrhea (8 mg/L). For a further study of the mechanism of this type of resistance, it is recommended that a molecular method should be used for the detection of the *mcr-1* and *mcr-2* genes; however, in our case, this has not been confirmed. To the best of our knowledge, COL resistance in companion animals has only been described in China [44], Germany [45], Finland [46], Ecuador [47] and the Netherlands [43]. COL is currently the last choice in the treatment of human infections caused by carbapenem-resistant enterobacteria.

The presence of tetracycline (TET) resistance was detected in the *E. coli* of both healthy (*n* = 13) and sick dogs (*n* = 14) with MIC 90 (32 mg/L). Similarly, relatively high levels of TET resistance have been documented in other studies of dogs; for example, in Italy, Belgium and the Netherlands [43] as well as in Poland [36]. In the past, tetracycline has been used not only to treat urinary tract infections (UTIs), but various derivates of TET (such as chlortetracycline) have been used as a growth promotor [48], and the resistance probably reflects the long history of this application. These results indicate that the resistance to TET is still growing, and it should be used only if the susceptibility of the bacteria is confirmed by an in vitro study. Resistance to TET is conferred by one or more of the described *tet* genes, which encode one of three resistance and efflux mechanisms that appear to be more abundant among Gram-negative microorganisms [49]. All of our isolates were examined for the presence of *tet*A and/or *tet*B genes. The most common determinant in the healthy isolates was the *tet*A gene (*n* = 19), while *tet*B was detected in five isolates. These results are comparable with others described by Costa et al., Torkan et al. and Yousefi et al. [50,51,52]. On the other hand, the isolates from dogs with diarrhea showed a higher prevalence of the *tet*B gene (*n* = 13) versus *tet*A (*n* = 5).

**Figure 1:**
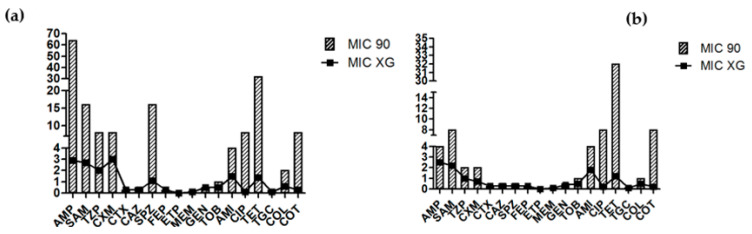
The values of MIC 90 and MIC XG (geometric mean MIC values of an antibiotic agent; mg/L) in *E. coli* of (**a**) healthy dogs and (**b**) dogs with diarrhea. Abbreviations: AMP = ampicillin; SAM = ampicillin + sulbactam; TZP = piperacillin + tazobactam; CXM = cefuroxime; CTX = cefotaxime; CAZ = ceftazidime; SPZ = cefoperazone + sulbactam; FEP = cefepime; ETP = ertapenem; MEM = meropenem; GEN = gentamicin; TOB = tobramycin; AMI = amikacin; CIP = ciprofloxacin; TET = tetracycline; TGC = tigecycline; COL = colistin and COT = trimethoprim + sulfonamide.

Fluoroquinolone resistance is multifactorial, with both chromosomal and plasmid-mediated quinolone resistance (PMQR) mechanisms that are often contributing to the overall MIC [53]. The emergence of PMQR indicates that quinolone resistance can also be acquired through a horizontal gene transfer [54], and PMQR genes can create an environment in *E. coli* for the rapid selection of high levels of resistance [55]. The MIC 90 of ciprofloxacin (CIP) was MIC 90 = 8 mg/L in both groups (Figure 1) and was higher than CBP (0.5 mg/L). Among the 38 healthy *E. coli* isolates, 16 carried PMQR genes including the *qnr*B gene in 13 isolates, *qnr*S in two isolates and one isolate with *aac*(6′)-*Ib-cr*. The isolates from dogs with diarrhea were positive for *qnr*S (*n* = 9) and *aac*(6′)-*Ib-cr* (*n* = 2). As in other studies [56,57], genes encoding PMQR were also present in the ciprofloxacin-sensitive isolates, and this was not only related to the selective pressure of the fluoroquinolones used.

Resistance to trimethoprim-sulphonamide (COT) was detected in 11 *E. coli* strains from the healthy dogs and 5 isolates from the diarrheal dogs. In this study, the trimethoprim determinant *dfr*A was harbored by three isolates of the healthy dogs. This rate of COT resistance gene acquisition is high, and may be due to selection resulting from the frequent use of the sulfonamide/trimethoprim combination (due to its broad-spectrum activity) in small animal medicine [51]. This may also explain the presence of *sul1* (*n* = 1 in the healthy dogs) and *sul*2 (*n* = 9 in the healthy dogs and *n* = 5 in the dogs with diarrhea) genes in our examined isolates. These results indicate a transmission of resistance genes to the normal microflora of healthy dogs.

Antimicrobial multidrug resistance (MDR) (resistance to at the least three different classes of antibiotics) was reported in 11 isolates of the healthy dogs and 2 isolates of the diarrheal dogs. The presence of integron 1 (*int1*; *n* = 12) and transposome (*tn3*; *n* = 12) in the healthy dogs indicates that the genetic mechanism for obtaining AMR genes is present not only in clinically-obtained isolates, but also in the isolates of a normal pet’s microbiota. The *int1* gene often occurs in combination with trimethoprim resistance (*dfr*) and resistance to sulphonamide (*sul*), and it was detected in two isolates from the healthy dogs.

Data on pet animals is clearly needed for guiding the antimicrobial use policies in small animal veterinary practice, as well as for assessing the risk of the transmission of antimicrobial resistance to humans. Although our work evaluated antibiotic resistance without comparing our isolates to human ones, there are other existing studies that provide support for the occasional cross-host-species sharing of resistant strains, which highlights the importance of understanding the role of companion animals in the overall transmission patterns of multi-drug resistant *E. coli* with the potential for causing intestinal and/or extraintestinal infection [58,59].

### 3.3. Phylogenetic Analysis and Biofilm Formation

**Figure 2:**
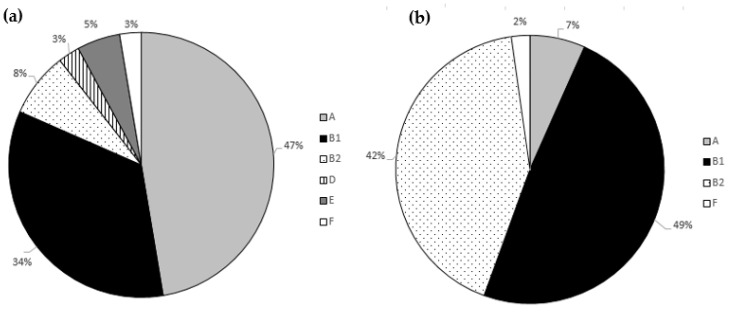
The *E. coli* phylogroup analysis in (**a**) healthy dogs and (**b**) dogs with diarrhea.

Focusing on the phylogenetic analysis (Figure 2), most of the strains from the healthy dog group were classified into commensal intestinal groups. In detail, 18 isolates were members of phylogroup A, and 13 were members of phylogroup B1. Pathogenic phylogroups occurred less frequently, but phylogroup B2 included three isolates; phylogroup E consisted of two isolates; one isolate fell into each of the phylogroup D and F groups.

The many strains from the dogs with diarrhea were classified into B2 (19/45; 42.2%) and B1 (22/45; 48.90%) groups. Our comparative analysis between the phylogroups of the healthy and diarrheic dogs showed that the phylogroup B2 was visibly more common in the dogs with diarrhea.

In the healthy animals, the B1 group predominated, followed by the A, B2 and D groups [60]. These findings are important and show that the healthy dogs are colonized by commensal and pathogenic strains. The observation that the phylogenetic group B2 was usually related with the uropathogenic *E. coli* (UPEC) infection and the phylogenetic group D with the other extraintestinal pathogenic *E. coli* (ExPEC) has been previously reported [61,62]. Our results are comparable with those of Vega-Manriquez et al. [63], where the phylogroup analysis showed that a greater half (57%) of the *E. coli* isolates from the healthy dogs belonged to the commensal A and B1 groups, in contrast to the sick dogs, where the phylogroups D and B2 were dominant. In a study by Valat et al. [64], most of the pathogenic *E. coli* in dogs from digestive pathologies were also assigned to the B2 phylogroup (58.6%).

**Figure 3:**
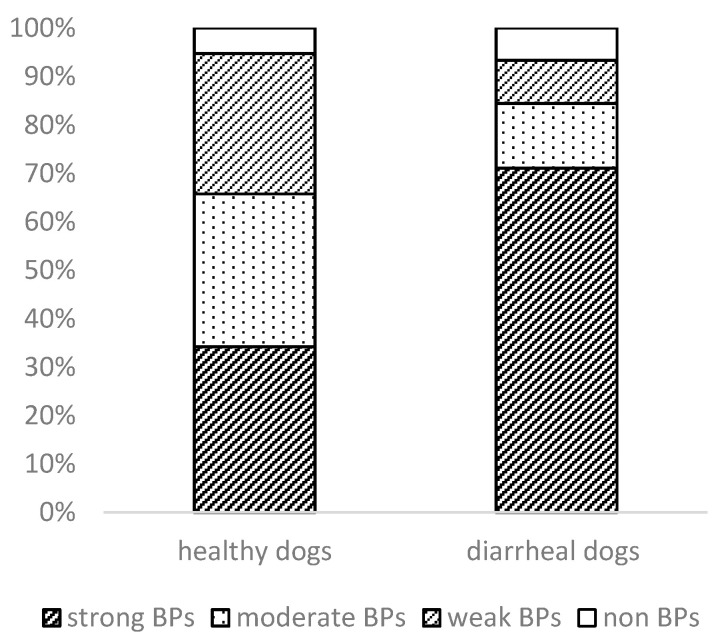
The ability of *E. coli* isolates to form biofilm.

The ability of *E. coli* to form a biofilm is an important virulent property. Our strains were divided into four main groups on the basis of their biofilm-producing capacity (Figure 3).

In the healthy dogs, 13 strains (34.2%) were classified as strong biofilm producers, while the remaining 12 strains (31.6%) were regarded as moderate and 11 (29%) as weak biofilm producers. Only two of the strains did not form a biofilm. Most of the clinical isolates (70%, *n* = 32) had a stronger ability to form biofilms, followed by 13% moderate and 9% weak biofilm producers. In their study, Vijay et al. [65] examined the ability to form a biofilm in enteroaggregative *E. coli* (EAEC) from humans and animals with diarrhea. In that case, the EAEC isolates recovered from animals were low biofilm producers (65.3%), followed by moderate (26.5%) and high biofilm producers (8.1%). It has been reported [66,67] that biofilm formation may be an important contributory factor in persistent infection, either by allowing the bacteria to evade the local immune system and/or by preventing the transport of antibacterial factors, including antibiotics.

The analysis between the phylogenetic groups and the presence of phenotypic AMR (Table 2) shows that 17 *E. coli* of the healthy dogs belonging to the commensal phylogenetic groups—A, B1—were without AMR phenotypic profile along with all examined strains belong to the pathogenic groups B2, D, E and F. The remaining 14 *E. coli*—part of the commensal phylogroups—showed resistance to antibiotics. The most common phenotypic AMR profile in the healthy dogs were AMP–TET–COT (phylogroup A = 2 isolates; B1 = 2 isolates) and AMP–CIP–TET–COT (phylogroup A = 2 isolates; B1 = 2 isolates). Twenty-three *E. coli* of the sick dogs belonging to the commensal phylogenetic groups— A, B1—were without AMR phenotypic profile, and two isolates showed phenotypic resistance only to colistin. Predominant isolates of dogs with diarrhea showed the most common form of CIP—TET combination in the B2 phylogroup (*n* = 7). Our study compared the values of MIC 90 and MIC XG (geometric mean MIC values of an antibiotic agent; mg/L) in *E. coli* of healthy dogs and dogs with diarrhea and points only to a slight increase in these values in healthy animals versus dogs with diarrhea.

## 4. Conclusions

This study reported on a comparison of *E. coli* isolates from healthy and diarrheic dogs. The observed results in the dogs with diarrhea showed differences in the phylogenetic representation, especially in terms of a high incidence of B2 isolates that were able to form a stronger biofilm compared to isolates from healthy dogs. The MIC 90 and MIC XG monitoring pointed out only a slight increase in these values in healthy animals. However, a high prevalence of genes encoding AMR and mobile elements in commensal *E. coli* can indicate that these strains can be a vehicle for the spread dissemination of AMR.

## Figures and Tables

**Table 1:** Primers used for the PCR detection of resistance genes and phylogroups.

Gene	Primer Sequences (5′–3′)	Annealing (°C)	Size Product (bp)	Reference
*int1*	F:GGGTCAAGGATCTGGATTTCG R:ACATGCGTGTAAATCATCGTCG	62	483	[13]
*tn3*	F:CACGAATGAGGGCCGACAGGA R:ACCCACTCGTGCACCCAACTG	58	500	[14]
*dfrA*	F:GTGAAACTATCACTAATGG R:TTAACCCTTTTGCCAGATTT	55	474	[15]
*dfrB*	F:GATCGCCTGCGCAAGAAATC R:AAGCGCAGCCACAGGATAAAT	60	141	[15]
*tetA*	F:GGCCTCAATTTCCTGACG R:AAGCAGGATGTAGCCTGTGC	55	372	[16]
*tetB*	F:GAGACGCAATCGAATTCGG R:TTTAGTGGCTATTCTTCCTGCC	55	228	[16]
*oqxA*	F:GACAGCGTCGCACAGAATG R:GGAGACGAGGTTGGTATGGA	62	339	[17]
*oqxB*	F:CGAAGAAAGACCTCCCTACCC R:CGCCGCCAATGAGATACA	62	240	[17]
*qepA*	F:GCAGGTCCAGCAGCGGGTAG R:CTTCCTGCCCGAGTATCGTG	60	199	[18]
*qnrS*	F:ACGACATTCGTCAACTGCAA R:TAAATTGGCACCCTGTAGGC	53	417	[19]
*qnrA*	F:ATTTCTCACGCCAGGATTTG R:GATCGGCAAAGGTTAGGTCA	53	516	[19]
*qnrB*	F:GATCGTGAAAGCCAGAAAGG R:ACGATGCCTGGTAGTTGTCC	53	469	[19]
*aac*(6′)-*Ib-cr*	F:GATCTCATATCGTCGAGTGGTGG R:GAACCATGTACACGGCTGGAC	58	435	[19]
*mcr-1*	F:CGGTCAGTCCGTTTGTTC R:CTTGGTCGGTCTGTAGGG	58	309	[20]
*mcr-2*	F: TGTTGCTTGTGCCGATTGGA R:AGATGGTATTGTTGGTTGCTG	58	567	[21]
*sul1*	F:CGGCGTGGGCTACCTGAACG R:GCCGATCGCGTGAAGTTCCG	69	433	[22]
*sul2*	F:GCGCTCAAGGCAGATGGCATT R:GCGTTTGATACCGGCACCCGT	69	293	[22]
*sul3*	F: GAGCAAGATTTTTGGAATCG R:CATCTGCAGCTAACCTAGGGCTTTGA	51	990	[23]
*arpA*	F:AACGCTATTCGCCAGCTTGC R:TCTCCCCATACCGTACGCTA	59	400	[12]
*chuA*	F:ATGGTACCGGACGAACCAAC R:TGCCGCCAGTACCAAAGACA	59	288	[12]
*yjaA*	F:CAAACGTGAAGTGTCAGGAG R: AATGCGTTCCTCAACCTGTG	59	211	[12]
*TspE4.C2*	F: CACTATTCGTAAGGTCATCC R: AGTTTATCGCTGCGGGTCGC	59	152	[12]
*arpAgpE*	F:GATTCCATCTTGTCAAAATATGCC R:GAAAAGAAAAAGAATTCCCAAGAG	57	301	[24]
*trpAgpC*	F:AGTTTTATGCCCAGTGCGAG R:TCTGCGCCGGTCACGCCC	59	219	[24]
*bla*_TEM-1_	F:ATGAGTATTCAACATTTCCG R:CCAATGCTTAATCAGTGAGG	55	858	[25]
*bla*_SHV_	F:ATGCGTTATATTCGCCTGTG R:TTAGCGTTGCCAGTGCTCGATG	58	301	[26]
cit	F: TGGCCAGAACTGACAGGCAAA R: TTTCTCCTGAACGTGGCTGGC	64	462	[27]

Abbreviations: *int1* = integron; *tn3* = transposon; resistance to trimethoprim = *dfrA*, *dfrB*; resistance to tetracycline = t*etA*, *tetB*; quinolone resistance = *oqxA*, *oqxB*, aac(6′*)-Ib-cr*, *qepA*, *qnrS*, *qnrA, qnrB*; resistance to colistin = *mcr-1*, *mcr-2*; sulfonamide resistance = *sul1*, *sul2* and *sul3*; β-lactamase encoding *bla*_TEM-1_, *bla*_SHV_ and ampicillinase–cit. Phylogenetic grouping: *arp*A, *chu*A, *yja*A, DNA fragment *TspE4.C2* and requires additional testing for specific genes in the E (*arpAgp*E) and C (*trpAgp*C) groups.

**Table 2:** The frequency of phenotypic antimicrobial resistance/sensitivity divided into phylogroups in healthy and sick dogs.

Phylogroups of Healthy Dogs	Phenotypic Antimicrobial Resistance Profile	Number of Isolates
A	Without AMR profile	*n* = 8
A	TET	*n* = 2
A	AMP, COT	*n* = 1
A	AMP, TET, COT	*n* = 2
A	AMP, SAM, TET	*n* = 1
A	AMP, CIP, TET, COT	*n* = 2
A	AMP, CIP, TET, COL, COT	*n* = 2
B1	Without AMR profile	*n* = 9
B1	AMP, TET, COT	*n* = 2
B1	AMP, CIP, TET, COT	*n* = 2
B2	Without AMR profile	*n* = 3
D	Without AMR profile	*n* = 1
E	Without AMR profile	*n* = 2
F	Without AMR profile	*n* = 1
Phylogroups of sick dogs	Phenotypic antimicrobial resistance profile	Number of isolates
A	Without AMR profile	*n* = 3
B1	Without AMR profile	*n* = 20
B1	COL	*n* = 2
B2	CIP	*n* = 4
B2	TET	*n* = 3
B2	CIP, TET	*n* = 7
B2	TET, COT	*n* = 2
B2	CIP, COT	*n* = 1
B2	SAM, TET, COT	*n* = 2
F	Without AMR profile	*n* = 1

Abbreviations: AMP = ampicillin; SAM = ampicillin + sulbactam; CIP = ciprofloxacin; TET = tetracycline; COL = colistin and COT = trimethoprim + sulfonamide.
